# Patient genetics shape the autoimmune response in the blistering skin disease pemphigus vulgaris

**DOI:** 10.3389/fimmu.2022.1064073

**Published:** 2023-01-10

**Authors:** John Baker, Kristina Seiffert-Sinha, Animesh A. Sinha

**Affiliations:** Dermatology Department, Jacobs School of Medicine and Biomedical Sciences, University at Buffalo, Buffalo, NY, United States

**Keywords:** pemphigus vulgaris, HLA, genetics, autoimmunity, ethnicity, autoantibodies

## Abstract

**Background and aim:**

Pemphigus vulgaris (PV) is known to have one of the strongest HLA associations among autoimmune diseases. DRB1*0402 and DQB1*0503 in particular are significantly overrepresented in PV patients in certain worldwide populations. Yet, there remain significant gaps in our understanding regarding the precise link between PV-associated HLA molecules, the specificity of the autoimmune response, and clinical expression. In this study we assessed correlations between factors including HLA genotype, ethnicity, autoantibody levels, and lesion distribution in a cohort of 293 patients.

**Methods and population:**

Participants were recruited from multiple outpatient dermatology clinic settings and patient support meetings in the USA. On intake, patients provided venous blood samples and answered questionnaires regarding their current disease activity.

**Results:**

Eighty-one percent of patients typed as either DRB1*0402 or DQB1*0503 with a high prevalence of DRB1*0402 in patients of Ashkenazi Jewish or Caucasian (non-Jewish) descent (86% and 42%, respectively) and DQB1*0503 in patients of Southeast Asian descent (78%). Patients typing as HLA DRB1*0402 had higher levels of anti-desmoglein (Dsg)3 antibodies (204.6 +/- 340.5 IU/ml) than patients without DRB1*0402 (138.5 +/- 236.4 IU/ml) (p=0.03) and had mucosal only lesions more often than cutaneous only or mucocutaneous lesions. Patients typing as DQB1*0503 had higher levels of anti-Dsg1 antibodies (47.3 +/- 59.8 IU/ml) compared to other groups (27.8 +/- 43.7 IU/ml) (p=0.06) and higher rates of mucocutaneous disease than other lesion types. We also report an unexpected HLA association of DRB1*0804 in PV patients of African descent. Sixty-four percent of this population carried the DRB1*0804 allele, and presented with highly elevated levels of anti-Dsg3 (p=0.02). However, neither African heritage nor the presence of DRB1*0804 correlated with a predilection to any specific lesion morphology. Patients that carried neither DRB1*0402, nor DQB1*0503 or DRB1*0804 had the lowest levels of anti-Dsg3 antibodies (60.0 +/- 80.0 IU/ml) and the highest rate of solely cutaneous disease compared to carriers of these alleles.

**Conclusion:**

Our data illuminate the broader impact of genetic factors on disease development by showing that differences in HLA expression among patients and ethnicities play a large role in driving distinct patterns of antibody selection and disease phenotype in PV. These findings provide insights regarding clinical heterogeneity, and are relevant to developing improved, patient tailored management strategies.

## Introduction

Pemphigus Vulgaris (PV) is a rare autoimmune blistering disorder characterized by mucosal and/or skin lesions due to the presence of autoantibodies against desmosomal proteins involved in cell-adhesion. Autoantibody binding leads to suprabasilar acantholysis in the epithelium resulting in superficial blisters that rupture easily and often present as open erosions. Like other autoimmune diseases, the etiology of PV is complex and multifactorial, involving genetic, environmental, and immunologic factors. Moreover, there is considerable clinical heterogeneity within given autoimmune diseases, including PV, that is unexplained and confounds clinical management. Numerous studies have reported a strong association between certain HLA alleles and PV, particularly the class II HLA alleles DRB1*0402 and DQB1*0503 (1). HLA alleles are major players in autoimmune development that predispose individuals to target self-antigens (2). These alleles are highly polymorphic genes of the major histocompatibility complex (MHC) coding for HLA class I and II molecules that are instrumental in antigen presentation for the initiation of immune responses, including those directed against self-antigens in the case of autoimmunity (3). While it is established that specific HLA alleles are a requirement for autoimmune development, the direct links between genetic susceptibility elements, the specificity of the autoimmune response and the ultimate clinical presentation of disease have not been clearly mapped.

There is some evidence in the literature indicating that carrying a particular HLA allele associated with a certain autoimmune condition may also influence disease presentation. For example, the DRB1*0103 allele is associated with the isolated colonic form of Crohn’s disease (4). In rheumatoid arthritis, a shared isotope involving 5 amino acids in the DRB1 locus is associated with earlier disease presentation and more severe bone erosions (5). For PV, the relationship between the expression of specific HLA alleles and disease presentation remains unclear, previous studies were limited by sample size and conflicting results (6–8). Svecova et al. reported that DRB1*0402 and DQB1*0302 were associated with more severe disease, but Le et al. conversely found that DRB1∗04 alleles are likely to be associated with mild and moderate disease. Notably these studies had no more than 50 PV patients per study, and the two conflicting studies are from Slovakia and India, two countries with relatively ethnically homogeneous populations.

Using a large biorepository of PV patients collected over two decades with well-annotated data on disease presentation, we set out to systematically assess for patterns linking a multitude of disease relevant parameters including ethnicity, HLA type, autoantibody levels, and lesion morphology. We confirm previously described HLA associations in well-studied ethnic populations, but also find an unexpected HLA association in the previously underrepresented group of PV patients of African descent. We also uncover associations of specific HLA allele expression with autoantibody profiles and phenotype. This information deepens our understanding of key pathogenetic mechanisms in PV that direct disease activity and underlie clinical heterogeneity, with relevance to improving management strategies targeted to patient subpopulations. This work illuminates previously unrecognized patterns linking genetic susceptibility, autoimmune specificity and disease phenotype.

## Materials and methods

### Patient population

Participants were enrolled into our autoimmune blistering disease biorepository *via* Dermatology outpatient clinics at Weill Cornell Medical College, Michigan State University, and the University at Buffalo, as well as annual meetings of the International Pemphigus and Pemphigoid Foundation (IPPF). The study was approved by the institutional review boards of all academic institutions involved (IRB number 0998-398, 05-1034, and 456887, respectively). All ethical guidelines were followed, including a written informed consent prior to enrollment.

Upon enrollment, patients with a biopsy confirmed diagnosis of PV provided information regarding demographics, disease history, lesion morphology, disease classification, family history, and past medical history. Patients also provided venous blood samples from which serum and DNA was isolated for immediate use or storage at -80°C for future use. Patients visiting more than once provided venous blood and current clinical information at subsequent visits when possible.

A total of 644 samples from 293 patients were included in this study. Patient demographic information can be found in **Table 1**. Pemphigus disease activity was determined using consensus guidelines created by the International Pemphigus Committee (9). Patients were considered to be active if they had 3 or more non-transient lesions (lasting longer than 1 week), experienced extension of existing lesions, or both.

**Table 1:** Study population demographics.

	Population (n)[# of samples]	Female/Male ratio	Mean Age of Onset (Years+/- SD)
*Total Patients*	*293 [647]*	*1.96*	*47.4 (+/- 13.6)*
African American	11 [35]	5	42.5 (+/- 13.5)
Ashkenazi Jewish	77 [269]	1.85	50.9 (+/- 12.6)
Caucasian	148 [259]	2.02	48.0 (+/- 14.0)
East Asian	6 [13]	1	39.5 (+/- 7.6)
Hispanic	28 [39]	2.5	44.3 (+/- 11.8)
South Asians	23 [32]	1.3	39.7 (+/- 14.1)

### Analysis of HLA

High resolution HLA typing was completed by PCR amplification using sequence specific primers at the Histocompatibility and Immunogenetics Laboratory at Michigan State University with commercial kits (One lambda, Thermo Fisher Scientific).

### Detection of anti-Dsg3 and anti-Dsg1

Anti-Dsg3 and anti-Dsg 1 levels were determined *via* ELISA (RG-M7593-D; MBL Intl., Woburn, MA) as per manufacturer’s protocol using a 1:101 serum dilution or diluted further as needed if the serum antibody levels were higher than 140 U/ml. The kits detect immunoglobulin G (IgG) antibodies directed against Dsg3 and Dsg1, but do not distinguish between IgG subclasses. Levels of anti-Dsg3 and -1 are presented in IU/ml. In order to determine whether a given unit value is considered positive, for the purposes of this publication, a cutoff of >20IU/ml was utilized as per previous manufacturer recommendations. However, it should be noted that the current manufacturer recommended cutoff for anti-Dsg 3/1 stands at 36/37 U/ml, but that our own research group considers a cut-off of 10 U/ml positive (based on comparisons with our own healthy control population).

### Detection of anti-thyroid peroxidase and anti-thyroglobulin levels

Anti-TPO ELISA and anti-Tg ELISA were completed *via* standard protocols using GenWay Biotech (San Diego, CA) kits (GWB-521202 and GWB-521201, respectively). These kits are able to detect immunoglobulin G (IgG) antibodies against TPO, but do not distinguish between subclasses of IgG. Anti-TPO positivity was defined as equal to or larger than 20IU/ml. This cutoff has been used in previous studies to determine anti-TPO positivity (10). Additionally, we determined that this cutoff resulted in 7.6% of our control population being positive for anti-TPO, which aligns with general findings that about 8% of healthy individuals present with anti-TPO positivity (11). Anti-Tg positivity was defined as equal to or larger than 5 IU/mL. This cutoff was determined statistically through our control subjects’ anti-Tg levels (mean and standard deviation (1.18 ± 2.03 IU/mL) and adding two standard deviations to the mean (5.24 IU/mL).

### Statistical analysis

One-way ANOVA testing was used to assess anti-Dsg3/-1 ELISA levels and anti-TPO/-Tg ELISA levels across ethnic groups and patients grouped by HLA association. P-values of p ≤ 0.05 were considered to be statistically significant. To assess frequencies of lesion morphologies across different ethnicities and HLA groups we employed chi-square test of independence to compare groups. To assess if individual patient populations had significant bias towards a specific lesion morphology, we used chi-square goodness of fit test. To determine our expected lesion distribution frequencies for the chi-square goodness of fit test we totaled all groups and used the distribution of lesion morphology as our expected frequencies.

## Results

### DRB1*0402, DQB1*0302, and DQB1*0503 are the most common PV-associated HLA alleles

It has previously been described that, after accounting for linkage disequilibrium, the truly disease-relevant HLA associations in PV are restricted to the DRB and DQB loci (1). To assess HLA DRB and DQB allele frequencies in our population, DRB and DQB alleles were first divided by allele group (e.g. 01+, 03+, 05+; low resolution typing). Certain HLA allele groups of interest were further separated by specific HLA protein expression typing if an association with PV had previously been suggested (e.g. DRB1*0402; high resolution typing).

**Figure 1:**
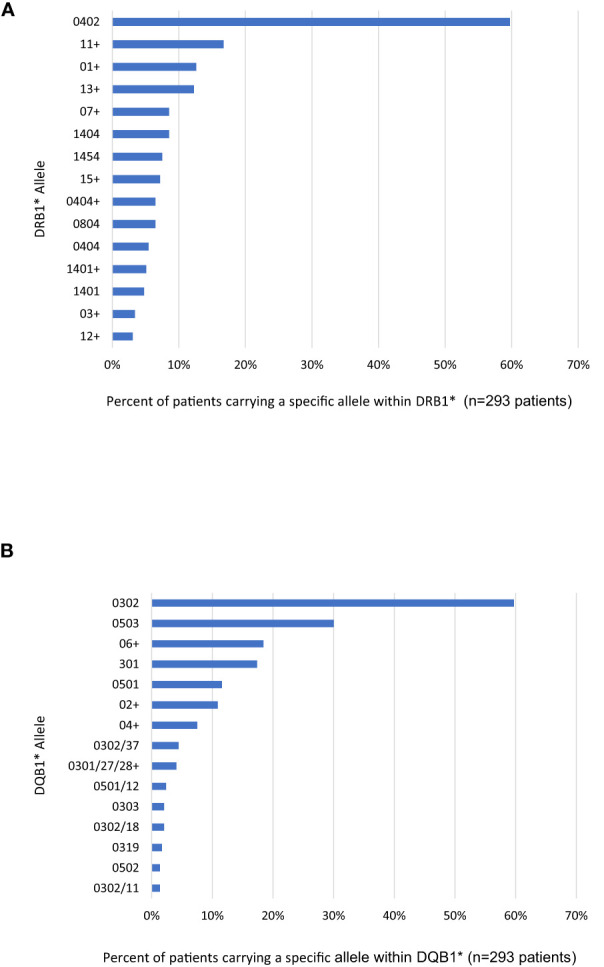
Percent of Patients Positive for most common HLA Alleles with **(A)** DRB and **(B)** DQB. Each patient expresses two alleles for DRB and DQB. The copy number of each allele was noted and allele frequencies were subsequently calculated as percent of patients carrying a given allele. The detected alleles are presented in descending order as allele group in low resolution (e.g. DRB1*01+ or DQB1*06+) if no specific HLA protein within that group was highly prevalent. If specific HLA alleles were highly prevalent within an allele group, they are presented in high resolution as the specific HLA protein (e.g. DRB1*04:02 and DRB1*04:04 or DQB1*03:02 and DQB1*03:03). The use of the symbol “/” indicated that the specific primers used for high resolution typing were unable to further distinguish between different alleles (i.e. DQB1*03:01/27/28 could be either DQB1*03:01, DQB1*03:27 or DQB1*03:28). The top 15 alleles or allele groups are shown in each locus.

The most common HLA allele within DRB1 was DRB1*0402 (60% of patients), distantly followed by DRB1*1404 (9%), DRB1*1454 (6%), and DRB1*0804 (6%) (**Figure 1A**). The most common specific DQB1 HLA alleles were DQB1*0302 (60% of patients) and DQB1*0503 (30%), followed by DQB1*0301 (17%), and DQB1*0501 (12%) (**Figure 1B**). Some of these alleles have previously been shown to be in linkage disequilibrium with each other, resulting in some non-pathogenic alleles commonly being present in pemphigus patients. For example, DQB1*0302 has been shown to be in linkage disequilibrium with DRB1*0402 and is not considered pathogenic for PV (1). Also, DRB1*1401, which was present in 2% of the population, has been shown to be in linkage disequilibrium with DQB1*0503 (1). It should also be noted that at the time of Lee et al. publication, DRB1*1454, present in 4% of patients, had yet to be discovered as a separate from DRB1*1401. Our data again confirms that DRB1*0402 and DQB1*0503 are the dominant alleles in pemphigus patients.

### Dominant PV-associated HLA allele expression varies between ethnic groups

**Figure 2:**
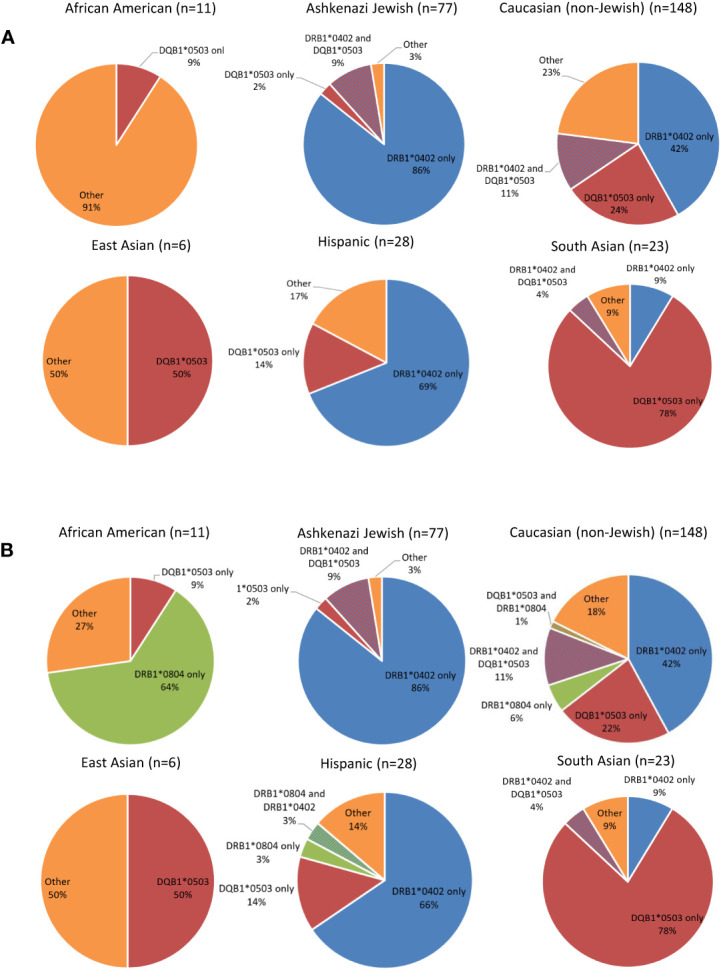
Distribution of PV-associated HLA alleles in PV Patients by Ethnicity. **(A)** Distribution of the known PV-associated HLA alleles DRB1*0402 and DQB1*0503 in PV patients by ethnicity. Expression of at least one allele of DRB1*0402 (blue) and DQB1*0503 (red) is expressed as percent out of the specific ethnic group analyzed. The percentage of patients not expressing either of these two alleles (“Other”) is depicted in orange. **(B)** Addition of DRB1*0804 expression to the distribution of the known PV-associated HLA Alleles DRB1*0402 and DQB1*0503 in PV Patients by Ethnicity. Expression of at least one allele of DRB1*0402 (blue), DQB1*0503 (red) and DRB1*0804 (green) is expressed as percent out of the specific ethnic group analyzed. The percentage of patients not expressing either of these three alleles (“Other”) is depicted in orange.

To assess whether HLA associations differed between ethnic groups, patients were stratified by ethnicity and assessed for presence or absence of the known pemphigus-associated HLA alleles DRB1*0402 and/or DQB1*0503 (**Figure 2A**). DRB1*0402 was the predominant allele in the Ashkenazi Jewish, and Hispanic populations and was also seen in half of the non-Jewish Caucasian population. The South Asian population was overwhelmingly positive for the presence of DQB1*0503 alleles, and 50% of East Asians that carried DQB1*0503 were DRB1*0402 negative. Interestingly, the African-American population was overwhelmingly negative for these two PV-associated HLA alleles, with only 1 patient (9% of patients) carrying DQB1*0503, and no patients carrying DRB1*0402. Upon assessing which HLA alleles other than DRB1*0402 and DQB1*0503 where present in the African-American population, we found that the DRB1*0804 allele was expressed in the majority of these patients (64%) (**Figure 2B**). The DRB1*0804 allele was also found in Caucasians and Hispanic patients at low rates (7% and 6%, respectively), and was not present in any other ethnicities, suggesting that this allele could be of disease relevance in the thus far understudied PV population of African descent.

### Autoantibody levels vary significantly with HLA status

**Figure 3:**
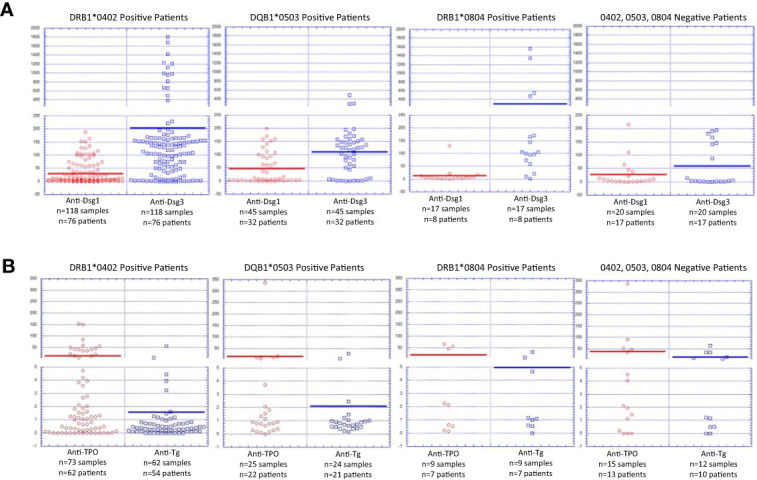
Autoantibody Levels by HLA Status in Active PV Patients. **(A)** Anti-Dsg1 and anti-Dsg levels by HLA association. Autoantibody levels are depicted for each patient carrying at least one allele of either DRB1*0402, DQB1*0503 or DRB1*0804 or not carrying either of these alleles. Anti-Dsg1 levels were highest in DQB1*0503 positive patients. Anti-Dsg3 levels were highest in patients carrying DRB1*0402 or DRB1*0804. Both anti-Dsg1 and anti-Dsg3 levels were lowest in patients that did not carry DRB1*0402, DQB1*0503, or DRB1*0804. **(B)** Anti-TPO and anti-Tg levels by HLA association. Autoantibody levels are depicted for each patient carrying at least one allele of either DRB1*0402, DQB1*0503 or DRB1*0804 or not carrying either of these alleles Both anti-TPO and anti-Tg levels were highest in patients without DRB1*0402, DQB1*0503, or DRB1*0804, and lowest in patients carrying DRB1*0402.

To assess whether autoantibody expression and levels were influenced by HLA association, patients in the active phase of disease (n=149 patients, 220 samples) were divided into groups based on HLA status and assessed for anti-Dsg serum profiles. In accordance with previous data by our group, anti-Dsg3 was the predominant antibody with higher mean serum levels detected across HLA-associations (12)(**Figure 3A**). DRB1*0804 positive patients had the highest average levels of anti-Dsg3 (298.3 +/- 459.3 IU/ml, 82% positive), followed by DRB1*0402-positive (204.6 +/- 340.5 IU/ml, 79% positive) and DQB1*0503-positive (111.4 +/- 94.7 IU/ml, 76% positive) patients. Patients that did not express either DRB1*0402, DQB1*0503, or DRB1*0804 had the lowest average anti-Dsg3 levels (60.0 +/- 80.0 IU/ml, 37% positive). Conversely, anti-Dsg1 antibody levels were highest in patients carrying the DQB1*0503 allele (47.3 +/- 59.8 IU/ml, 42% positive), while DRB1*0804 patients had the lowest average anti-Dsg1 levels (13.4 +/- 30.5 IU/ml, 12% positive) (**Figure 3A**). Overall, differences in anti-Dsg3 levels reached statistically significant levels with a p value of p=0.03363 (one-way ANOVA), while variability in anti-Dsg1 levels only approached statistical significance with p=0.05963.

In addition to anti-Dsg3 and 1 reactivity, other autoantibodies have been described in patients with PV (13–15). Among these non-Dsg antibodies, anti-thyroid peroxidase (TPO) and anti-thyroglobulin (Tg) reactivity has been found at higher rates in PV patients than in healthy controls (14, 15) and that we have previously described anti-TPO reactivity is driven by HLA status (14). Thus, we also assessed anti-TPO and anti-Tg reactivity in active patients divided by HLA-status (**Figure 3B**). We find that both anti-TPO and anti-Tg levels were highest in patients without DRB1*0402, DQB1*0503, or DRB1*0804 at 38.2 +/- 86.6 IU/ml (33% positive) and 13.3 +/- 19.8 IU/ml (50% positive) respectively, while DRB1*0402 positive patients had the lowest average levels of both with mean anti-TPO of 12.6 +/- 29.3 IU/ml (19% positive) and mean anti-Tg of 1.6 +/- 7.1 IU/ml (3% positive). Differences in average anti-Tg levels between HLA groups were found to be statistically significant with p=0.00141 on one-way ANOVA.

### Anti-Dsg3/1 levels vary significantly with ethnicity

It has been previously observed that autoantibody levels may vary between patients of Indian origin vs. white northern Europeans (16). To further assess whether autoantibody expression and levels were influenced by ethnicity, we divided patients in the active phase of disease (n=156 patients, 224 samples) by ethnicity and assessed the respective anti-Dsg and anti-thyroid autoantibody serum profiles for each group.

**Figure 4:**
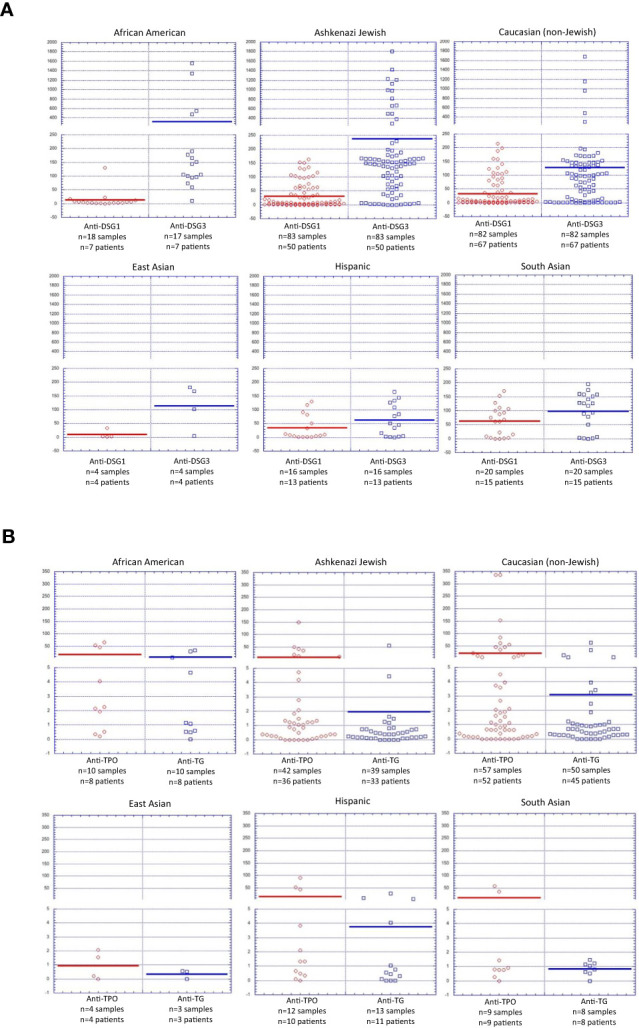
Autoantibody Levels by Ethnicity in Active PV Patients. **(A)** Anti-Dsg1 and Anti-Dsg3 antibody levels by ethnicity depicted for each patient within a specific ethnic group. **(B)** Anti-TPO and Anti-Tg antibody levels by ethnicity depicted for each patient within a specific ethnic group.

The highest levels of anti-Dsg3 Abs were found in African American patients (317.5 +/- 449.7 IU/ml, 94% positive), followed by the Ashkenazi Jewish (237.7 +/- 351.2 IU/ml, 83% positive) and Caucasian population (127.6 +/- 224.3 IU/ml, 67% positive). Anti-Dsg3 levels were lowest in Hispanic patients (62.4 +/- 58.3 IU/ml, 63% positive) (**Figure 4A**). Conversely, South Asian patients displayed the highest average anti-Dsg1 levels (63.7 +/- 54.7 IU/ml, 65% positive), while East Asian, with 10.2 +/- 15.2 IU/ml (25% positive), and African American, with 12.9 +/- 29.7 (11% positive), patients had the lowest average anti-Dsg1 levels (**Figure 4A**). Both anti-Dsg3 and anti-Dsg1 levels were found to vary significantly between ethnic groups, with p values of p=0.01763 and p=0.03258 respectively (one-way ANOVA).

Mean anti-TPO levels ranged from 8 to 23 IU/ml between ethnicities, with the notable exception of East Asian patients for which the mean was less than 1 IU/ml (0% positive). East Asian patients also had the lowest average levels for Anti-Tg, 0.35 +/- 0.3 IU/ml (0% positive), however, this finding is of limited significance due to the low number of East Asian patients (n=6) enrolled in this study. African American patients had the highest average Anti-Tg (7.7 +/- 12.6, 30% positive) (**Figure 4B**). Interestingly, of all DQB1*0503 positive PV patients, South Asian patients display higher anti-Dsg1 autoantibody levels (mean 66.5 +/- 56.9 IU/ml, 63% positive) than DQB1*0503 positive patients of all other ethnicities combined (36.7 +/- 59.7 IU/ml, 31% positive)(**Supplemental Figure 1**), indicating that both HLA expression as well as ethnicity can be considered independent risk factors for development of anti-Dsg1 autoantibodies.

### Predominant lesion morphology varies by HLA type and ethnicity

**Figure 5:**
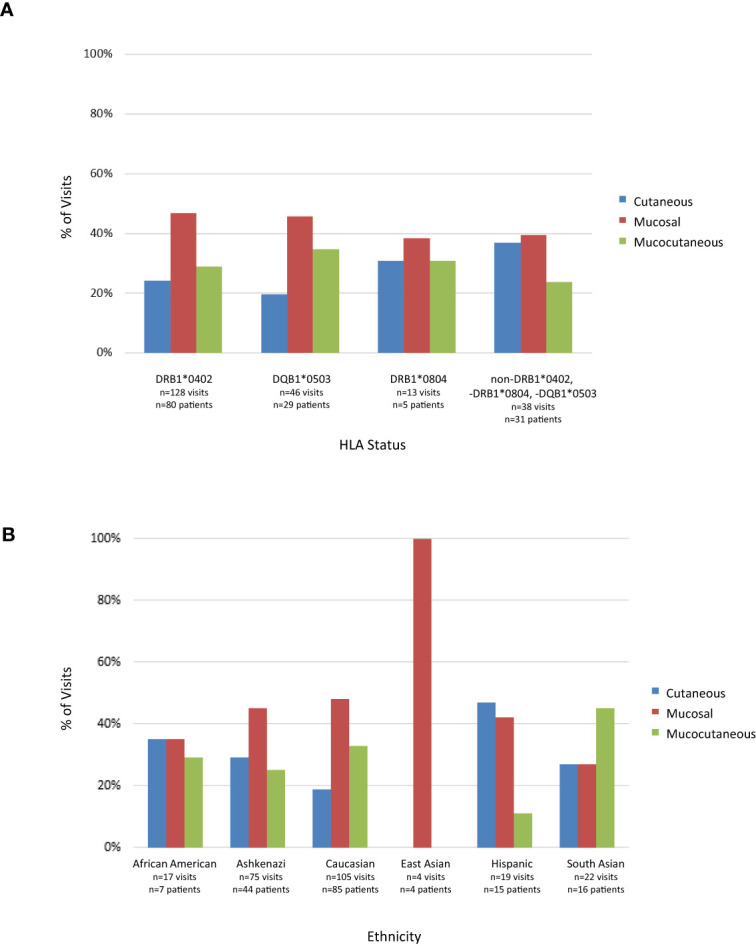
Lesion Morphology at time of visit in active PV patients. **(A)** Lesion morphology by HLA association. Lesion morphology was assessed for each patient carrying at least one allele of either DRB1*0402, DQB1*0503 or DRB1*0804 or not carrying either of these alleles at time of visit when in active disease. **(B)** Lesion morphology by ethnicity. Lesion morphology was assessed for each visit of patients within a specific ethnic group in the active phase of disease. Note that some patients were seen several times over the course of their study participation.

To assess patterns of lesion morphology within HLA groups, we stratified patients into groups based on HLA status (DRB1*0402 positive, DQB1*0503 positive, DRB1*0804 positive, and patients lacking expression of DRB1*0402, DRB1*0804, or DQB1*0503) and recorded lesion morphology as mucosal only, mucocutaneous or cutaneous only at time of blood draw. Lesion morphology tended to display similar patterns across HLA types with mucosal only being the dominant lesion morphology throughout all groups. Mucocutaneous morphology followed solely mucosal presentation in all groups possessing the DRB1*0402, DQB1*0503, or DRB1*0804 alleles. However, patients that did not carry the DRB1*0402, DQB1*0503, or DRB1*0804 alleles were the only group to have more visits with solely cutaneous lesions than mucocutaneous lesions (**Figure 5A**).

In light of the association between HLA expression and ethnicity in PV shown above, we assessed whether the differences in lesion morphology seen within HLA subgroups extend to different ethnic groups. Mucosal lesions were the predominant manifestation of PV for patients of Caucasian and Ashkenazi Jewish descent. East Asians displayed only mucosal disease at time of intake, although conclusions cannot be drawn at this time due to the small sample size. Solely cutaneous lesions were equal to or more common than mucosal only in the African American, South Asian and Hispanic populations. South Asians were the only population to have a predominant mucocutaneous presentation at visits (**Figure 5B**). Taken together these data indicate that morphology is skewed towards mucosal presentation in Jewish and non-Jewish Caucasian populations, while it is skewed towards cutaneous presentation in the Hispanic population and is equally distributed among South Asians and African Americans. Chi-squared goodness of fit test was used to assess variation in lesion morphology by ethnicity and HLA group (see Materials and Methods). The only population that approached statistical significance was the Hispanic population (p=0.06). While the East Asian population had a p-value approaching significance (=0.087), there were only four samples in this group, limiting the significance of this finding.

### No association of PDAI scores with gender, age of onset, ethnicity, and HLA

PDAI scores were available for a small subset of our study population visits (n=85). PDAI values by gender, age of onset, HLA, and ethnicity can be found in **Supplementary Table 1**. We found no statistically significant differences regarding PDAI values and gender, age of onset, ethnicity, and HLA status. Future studies with larger numbers of patients will be needed to better assess the extent to which genetic factors impact overall disease severity.

## Discussion

It is understood that HLA molecules play a crucial role in the HLA-peptide-TCR tripartite that is crucial for mounting adaptive immune system responses to both foreign pathogens as well as self-tissue targets in the case of autoimmune disease (17). By directing the autoimmune response and the effector cells and autoantibodies that drive it, we speculate that HLA alleles also help shape the clinical presentation brought on by these antibodies.

Our data confirms and expands on previously determined associations between specific HLA alleles and PV. For instance, for DRB1, we confirm the well-known association of the 0402 allele and PV (1) in the larger study population analyzed here, where DRB1*0402 is the most common pemphigus-associated allele. The second most common specific DRB1 allele in our population was found to be 1404, which has been suggested as possibly associated with PV in Chinese and Pakistani studies, but its significance has yet to be determined (18, 19). In our population, DRB1*0402 is paired with DQB1*0302 in almost all cases (172 out of 175 total). Our group has previously shown that DQB1*0302 is most likely in itself not a major contributor to disease, but is inherited together with DRB1*0402 because of strong linkage disequilibrium (1). Following DQB1*0302, we found DQB1*0503 as the most common DQB allele, which has been reported for PV previously (1).

It is striking, however, how the distribution of these two PV-associated HLA alleles varies between ethnic groups. PV patients identifying as being of Ashkenazi Jewish descent are predominant carriers of the DRB1*0402 allele (95% of patient within this ethnic group carry at least one DRB1*0402 allele). This is in line with (i) studies on Jewish PV patients from Israel that report around 90% DR4 positivity (20, 21) and (ii) the reported allele frequency of up to 15% of this allele in the general Israeli population (22, 23). Despite the high prevalence of DRB1*0402 in this population, PV is still rare within Israel, highlighting the multifactorial genesis of PV in which other factors in addition to the HLA background ultimately led to disease expression.

DQB1*0503, on the other hand, is the predominant allele in the South Asian population (in our study 82% of all South Asian patients carry this allele). An overabundance of this allele has been shown in this population before (allele frequency of up to 13% in a study from India) (24, 25). Despite the fact that both DRB1*0402 and DQB1*0503 are fairly rare amongst European populations, these are the two leading alleles within non-Jewish Caucasian PV patients who show a fairly equal distribution of DRB1*0402 and DQB1*0503 (55% and 36% of all non-Jewish Caucasian patients, respectively).

In addition to the accepted HLA alleles, our study has uncovered a previously underappreciated allele in PV, namely DRB1*0804. While only 6% of our entire patient population are DRB1*0804 positive, it should be noted that this allele is most commonly seen in African American PV patients, of which 64% are carriers. Since only one of these African-American patients carried the previously PV-associated allele DQB1*0503 and none carried DRB1*0402, the data suggest that DRB1*0804 can be considered a PV susceptibility allele in its own right. DRB1*0804 has been associated with African descent in several studies (26, 27). A comprehensive analysis of HLA allele frequencies worldwide found that DRB1*0804 is by far the most frequent in northwestern Africa, around modern Burkina Faso, and is more prevalent throughout Africa than other continents (24). While DRB1*0804 has not been studied as extensively in the context of PV, previous studies have noted that DRB1*0804 is increased in PV patients in Brazil and Egypt (28, 29).

Our findings highlight how closely PV is associated with different HLA alleles in different ethnic populations. Importantly, beyond establishing associations of HLA expression in PV patients of different ethnic backgrounds, our data further shows that the expression of certain HLA molecules in the context of PV is highly associated with, and likely shapes antibody selection and ultimately lesional phenotype. In this context, it has previously been shown that HLA DRB1*0402 contains a binding motif that can selectively bind Dsg3 specific peptides (30) and present them to DRB1*0402-restricted autoreactive CD4(+) T cells to initiate the production of Dsg3-reactive IgG autoantibodies (31). In line with these findings, patients carrying DRB1*0402 were found to express relatively higher levels of anti-Dsg3 autoantibodies. DRB1*0402 positive patients, including those with ethnicities in which the allele is prevalent, such as Ashkenazi Jewish and non-Jewish Caucasian, were predisposed to developing mucosal PV lesions over mucocutaneous and cutaneous only lesions.

Patients that carried DQB1*0503, including South Asian patients who are heavily DQB1*0503 positive, had higher rates of mucocutaneous disease than other patients. Moreover, expression of the DQB1*0503 allele was found to predispose patients to having relatively higher levels of anti-Dsg1 autoantibodies, perhaps a reflection of differing peptide binding capacities between DQB1*0503 vs DRB1*0402 molecules that link to anti-Dsg3 and anti-Dsg1 responses. Interestingly, when DQB1*0503 positive patients were subdivided by ethnicity, South Asian patients (n=16) had average anti-Dsg1 levels almost twice that of patients of other ethnicities (n=30) (**Supplemental Figure 1**), indicating that South Asian ethnicity, beyond reasons linked to HLA type, in itself is associated with elevated anti-Dsg1 expression, implicating additional thus far unknown genetic and/or environmental factors. While these results did not reach statistical significance (p=0.11), repeating this analysis with a larger sample size may elucidate the true significance of this discrepancy. Nevertheless, our findings are consistent with a study by Harman et al. in which 75% of Indian PV patients were positive for anti-Dsg1, compared to only 45% of White patients and 56% of patients of “other” ethnic background (16).

It is noteworthy that patients typing as DRB1*0804, despite displaying the highest mean levels of anti-Dsg3 and the lowest mean levels of anti-Dsg1 when compared to all other HLA-associations, did not show a clear predilection for morphology. In fact, this population split equally into patients carrying mucosal and patients carrying cutaneous lesions, indicating that autoantibody selection in itself it not an absolute factor in determining lesion location (skin vs. mucosal). This point is further underlined by the fact that the Hispanic population, despite being predominantly DRB1*0402 positive, displays relatively low levels of anti-Dsg3 and also did not have a predisposition towards mucosal disease, but instead was the only population with more cutaneous disease than mucosal or mucocutaneous. These data indicate that while DRB1*0402 appears to favor the selection of anti-Dsg3 antibody specificities, correspondingly leading to the predominantly mucosal pattern predicted according to the Dsg-Compensation hypothesis (32, 33), supplemental genetic and/or environmental elements must also play a role in determining lesional fate, again underscoring the multifactorial basis of disease.

Of note, patients that did not express the typical disease-associated HLA alleles, i.e. those typing negative for DRB1*0402, DQB1*0503, or DRB1*0804, had very low levels of anti-Dsg antibodies and the highest levels of cutaneous only lesions among all HLA-subgrouping analyzed. We and others have suggested before that non-Dsg antibodies or other thus far underappreciated factors may help explain lesion development in the absence of the accepted PV-associated antibodies (13, 34–36). Our group has also previously reported that in a slightly smaller patient set than the one used in this study, anti-TPO and anti-Tg have higher activity rates in patients not expressing DRB1*0402 and/or DQB1*0503. This study supports the notion that non-Dsg antibodies such as those directed against anti-TPO and-Tg may be of relevance in patients lacking the typical PV-associated alleles, since we show the highest levels of both antibodies in either DRB1*0804 positive patients or those lacking all 3 susceptibility alleles (DRB1*0402-, DQB1*0503- and DRB1*0804-triple negative). It should be noted that a number of non-Dsg autoantibodies besides anti-TPO and anti-Tg have been reported in PV (13, 35). While not investigated in this report, these specificities may similarly play a role in fine-tuning the autoimmune response in select patients.

Overall, our analysis reveals and reinforces an overarching theme: there are clear variances in HLA types found in PV patients of differing ethnic origin (including an unexpected and novel HLA association in PV patients of African descent, a historically understudied population in the context of PV), and these variances in HLA type link to variances in autoantibody profiles and clinical morphology. Taken together, our data provide strong support for the novel hypothesis that HLA allele expression not only predisposes to disease development in PV, but is also crucial in shaping autoantibody profiles and phenotypic expression, and that HLA variance undergirds disease heterogeneity. We report, for the first time, clear distinctions regarding associations of various disease associated HLA alleles (which are unevenly distributed across various ethnic groups) with key immunological and clinical parameters of disease (**Table 2A**, **Supplemental Figure 2A**). While the relative contributions of as yet unknown genetic and environmental factors to autoimmune activity will need further investigation, several previously unrecognized patterns and trends linked to HLA type have emerged (**Table 2B**, **Supplemental Figure 2B**): 1) Patients typing as HLA DRB1*0402 (found most commonly in Ashkenazi Jewish and Caucasian non-Jewish populations) had higher levels of anti-Dsg3 antibodies than patients without DRB1*0402 and had mucosal only lesions more often than cutaneous only or mucocutaneous lesions. 2) Patients typing as DQB1*0503 (found most commonly in patients of East Asian and South Asian descent) had higher levels of anti-Dsg1 antibodies compared to other groups and higher rates of mucocutaneous disease than other lesion types. 3) PV patients of African descent carried predominantly the DRB1*0804 allele, presented with highly elevated levels of anti-Dsg3, but did not link to any specific lesion morphology. 4) Patients that carried neither DRB1*0402, nor DQB1*0503 or DRB1*0804 had the lowest levels of anti-Dsg3 antibodies and the highest rate of solely cutaneous disease compared to carriers of these alleles.

**Table 2A:** Summary of relationships between ethnicity, HLA type, autoantibody profile, and lesion morphology.

Ethnicity	HLA Predominance	Autoantibodies				Clinical Presentation		
		Anti-Dsg3*	Anti-Dsg1*	Anti-TPO	Anti-Tg	Mucosal	Mucocutaneous	Cutaneous
African American	DRB1*0804	Very High	Very Low	High	Very High	35%	29%	35%
Ashkenazi	DRB1*0402	Very High	Low	Average	Low	45%	25%	29%
Caucasian	DRB1*0402 > DQB1*0503	High	Low	High	High	48%	33%	19%
East Asian	DQB1*0503	Average	Low	Very Low	Low	100%	0%	0%
Hispanic	DRB1*0402 > DQB1*0503	Low	High	Average	High	42%^⌂^	11%^⌂^	47%^⌂^
South Asian	DQB1*0503	Average	Very High	Average	Low	27%	45%	27%

The symbol * indicates the finding was of statistical significance with p<0.05. The symbol ^⌂^ indicates that the finding approached statistical significance with p ≤ 0.06. Any categories without either of these two symbols represent trends observed that did not meet statistical significance.

**Table 2B:** Summary of relationships between HLA type, autoantibody profile, and lesion morphology.

HLA Type	Autoantibodies				Clinical Presentation		
	Anti-Dsg3*	Anti-Dsg1^⌂^	Anti-TPO	Anti-Tg*	Cutaneous	Muco-cutaneous	Mucosal
DRB1*0402	**+++**	**++**	**+**	**-**	**+**	**+**	**+++**
DQB1*0503	**++**	**+++**	**++**	**+**	**+**	**++**	**+++**
DRB1*0804	**+++**	**-**	**++**	**+++**	**++**	**++**	**++**
Non-DRB1*0402, DQB1*0503, DRB1*0804	**-**	**++**	**+++**	**+++**	**++**	**+**	**++**

The symbol * indicates the finding was of statistical significance with p<0.05. The symbol ^⌂^ indicates that the finding approached statistical significance with p<0.06. Relative levels of lesion morphology and antibody levels have been displayed from absent to very elevated via symbols of -, +, ++, and +++ respectively. Any categories without either of these two symbols represent trends observed that did not meet statistical significance.

Interestingly, PDAI scores available for a limited number of patient visits did not reveal any significant differences when analyzed by gender, age of onset, HLA, or ethnicity, suggesting that disease severity is not predetermined by any of these factors individually. However, PDAI scores were not available for all patients or all visits and will have to be reassessed in a larger patient population later.

Future studies will be needed to elucidate the details of (auto)antigen recognition by each PV linked HLA allele that drive T/B cell activation and autoantibody selection and, in turn, disease morphology (lesion location). Defining these connections can be expected to deliver a fuller understanding of autoimmune induction and disease manifestation in pemphigus, and will advance the path towards precision medicine where individual genetic and immune response elements are incorporated into clinical decision making and patient management. Detailed information on the mechanisms by which HLA genes direct and determine disease-defining features is required to unravel the complexities of disease heterogeneity can be expected to improve the ability to diagnose, track and treat.

## Data availability statement

The raw data supporting the conclusions of this article will be made available by the authors, without undue reservation.

## Ethics statement

The studies involving human participants were reviewed and approved by Institutional Review Board, University at Buffalo Institutional Review Board, Michigan State University Institutional Review Board, Weill Medical College at Cornell University. The patients/participants provided their written informed consent to participate in this study.

## Author contributions

AS and KS-S devised the project and performed data collection. JB performed data selection, data analysis. All authors contributed to writing and critical revision of the manuscript.

## Supplementary material

Supplementary Figure 1 Anti-Dsg1 levels in DQB1*0503-carrying South Asian patients compared to all other DQB1*0503-carrying Populations. South Asian patients carrying DQB1*0503 (n=16) had nearly twice the average anti-Dsg1 (mean 66.5 +/- 56.9 IU/ml) level than DQB1*0503 patients of all other ethnicities combined (n=30) (36.7 +/- 59.7 IU/ml). However, this difference was not found to be statistically significant (p=0.11, unpaired t-test).

Supplementary Figure 2
**(A)** Summary Figure of Ethnicity Associations. Autoantibody levels and dominant lesion morphology associated with six ethnicity categories analyzed for this manuscript: African American, Ashkenazi Jewish, non-Jewish Caucasian, East Asian, Hispanic, and South Asian. Relative levels of autoantibodies and lesion morphologies are displayed by vertical bars, with increased height correlating to higher antibody levels. The symbol * indicates the finding was of statistical significance with p<0.05. The symbol ⌂ indicates that the finding approached statistical significance with p ≤ 0.06. Any categories without either of these two symbols represent trends observed that did not meet statistical significance. **(B)** Summary Figure of HLA Associations. Autoantibody levels and dominant lesion morphology associated with the four major HLA categories analyzed for this manuscript: DRB1*0402 positive, DQB1*0503 positive, DRB1*0804 positive, and those negative DRB1*0402, DQB1*0503, and DRB1*0804. Relative levels of autoantibodies and lesion morphologies are displayed by vertical bars, with increased length correlating to higher antibody levels. The symbol * indicates the finding was of statistical significance with p<0.05. The symbol ⌂ indicates that the finding approached statistical significance with p ≤ 0.06. Any categories without either of these two symbols represent trends observed that did not meet statistical significance.

Supplementary Table 1 PDAI values by gender, HLA, ethnicity, and age of onset. Within each category, the subgroups were compared among each other using one-way ANOVA. No statistically significant differences were found in any category.
