# Supplementary figures and images for: Patient genetics shape the autoimmune response in the blistering skin disease pemphigus vulgaris
Source: Front Immunol. 2023 Jan 10;13:1064073. doi: 10.3389/fimmu.2022.1064073 (PMC9871500; doi:10.3389/fimmu.2022.1064073)

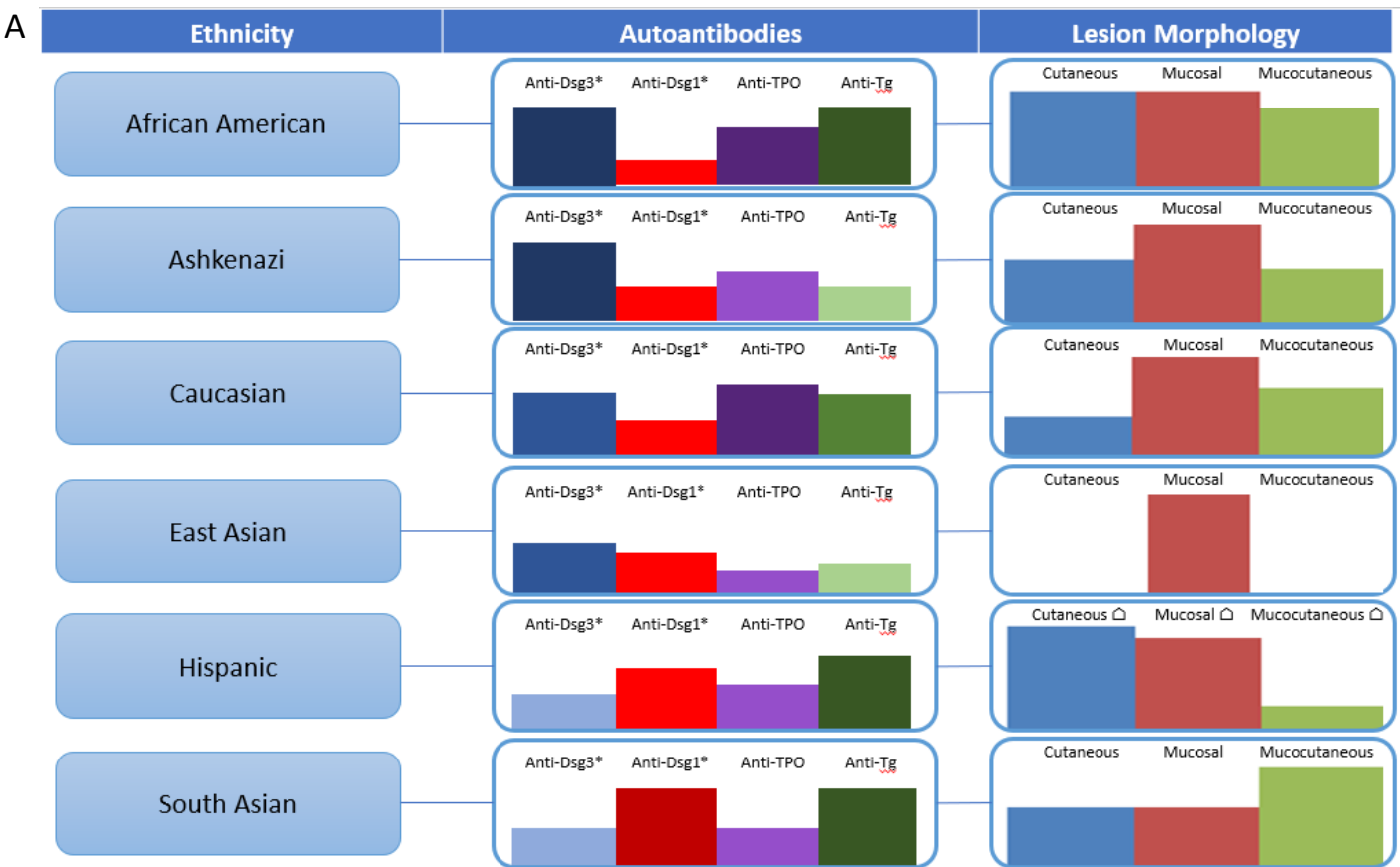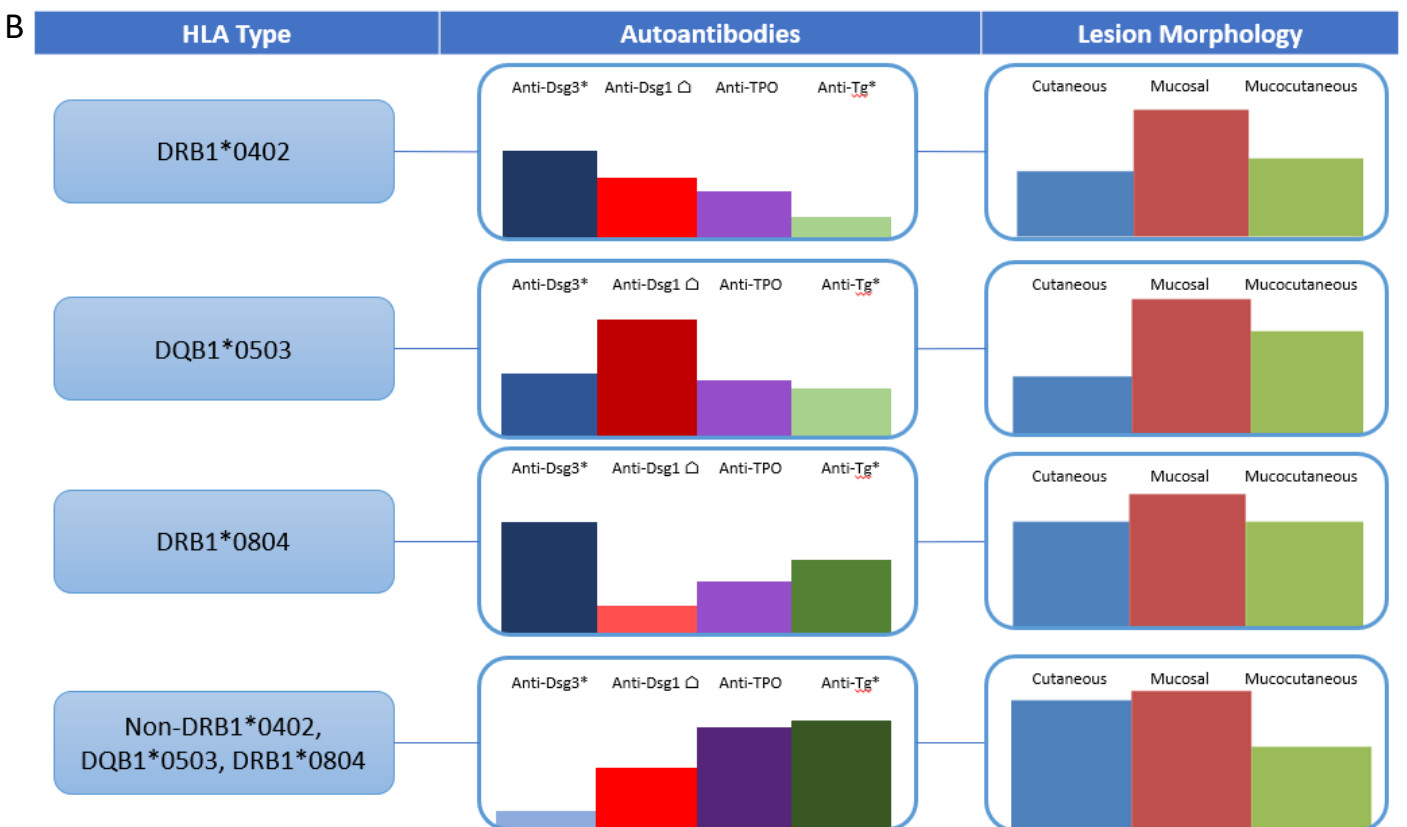

Supplement: Supplementary Figure 2 — (A) Summary Figure of Ethnicity Associations. Autoantibody levels and dominant lesion morphology associated with six ethnicity categories analyzed for this manuscript: African American, Ashkenazi Jewish, non-Jewish Caucasian, East Asian, Hispanic, and South Asian. Relative levels of autoantibodies and lesion morphologies are displayed by vertical bars, with increased height correlating to higher antibody levels. The symbol * indicates the finding was of statistical significance with p<0.05. The symbol ⌂ indicates that the finding approached statistical significance with p ≤ 0.06. Any categories without either of these two symbols represent trends observed that did not meet statistical significance. (B) Summary Figure of HLA Associations. Autoantibody levels and dominant lesion morphology associated with the four major HLA categories analyzed for this manuscript: DRB1*0402 positive, DQB1*0503 positive, DRB1*0804 positive, and those negative DRB1*0402, DQB1*0503, and DRB1*0804. Relative levels of autoantibodies and lesion morphologies are displayed by vertical bars, with increased length correlating to higher antibody levels. The symbol * indicates the finding was of statistical significance with p<0.05. The symbol ⌂ indicates that the finding approached statistical significance with p ≤ 0.06. Any categories without either of these two symbols represent trends observed that did not meet statistical significance. [file Image_2.pdf]
