# Supplementary material for: Patient genetics shape the autoimmune response in the blistering skin disease pemphigus vulgaris
Source: Front Immunol. 2023 Jan 10;13:1064073. doi: 10.3389/fimmu.2022.1064073 (PMC9871500; doi:10.3389/fimmu.2022.1064073)
Supplement: Supplementary Table 1 — PDAI values by gender, HLA, ethnicity, and age of onset. Within each category, the subgroups were compared among each other using one-way ANOVA. No statistically significant differences were found in any category. [file Table_1.docx]

# Supplementary Tables

| **PDAI by Gender** | PDAI Score (mean ± SD) |
| --- | --- |
| Female (n=51) | 7 ± 8 |
| Male (n=34) | 8 ± 11 |
| **PDAI by HLA** |  |
| DRB1*0402 (n=47) | 6 ± 5 |
| DQB1*0503 (n=21) | 10 ± 9 |
| DRB1*0804 (n=4) | 10 ± 15 |
| Other HLA (n=13) | 6 ± 5 |
| **PDAI by Ethnicity** |  |
| African American (n=3) | 8 ± 7 |
| Ashkenazi (n=29) | 5 ± 5 |
| Caucasian (n=38) | 9 ± 11 |
| East Asian (n=2) | 3 ± 3 |
| Hispanic (n=6) | 4 ± 4 |
| South Asian (n=11) | 9 ± 13 |
| **PDAI by Age of Onset** |  |
| Under 30 (n=6) | 9 ± 7 |
| 30-39 (n=14) | 8 ± 11 |
| 40-49 (n=31) | 6 ± 5 |
| 50-59 (n=17) | 6 ± 6 |
| Over 60 (n=15) | 7 ± 7 |

**Supplemental Table 1.** PDAI values by gender, HLA, ethnicity, and age of onset. Within each category, the subgroups were compared among each other using one-way ANOVA. No statistically significant differences were found in any category.
