# Supplementary material for: Patient genetics shape the autoimmune response in the blistering skin disease pemphigus vulgaris
Source: Front Immunol. 2023 Jan 10;13:1064073. doi: 10.3389/fimmu.2022.1064073 (PMC9871500; doi:10.3389/fimmu.2022.1064073)
Supplement: Supplementary Figure 1 — Anti-Dsg1 levels in DQB1*0503-carrying South Asian patients compared to all other DQB1*0503-carrying Populations. South Asian patients carrying DQB1*0503 (n=16) had nearly twice the average anti-Dsg1 (mean 66.5 +/- 56.9 IU/ml) level than DQB1*0503 patients of all other ethnicities combined (n=30) (36.7 +/- 59.7 IU/ml). However, this difference was not found to be statistically significant (p=0.11, unpaired t-test). [file Image_1.pdf]

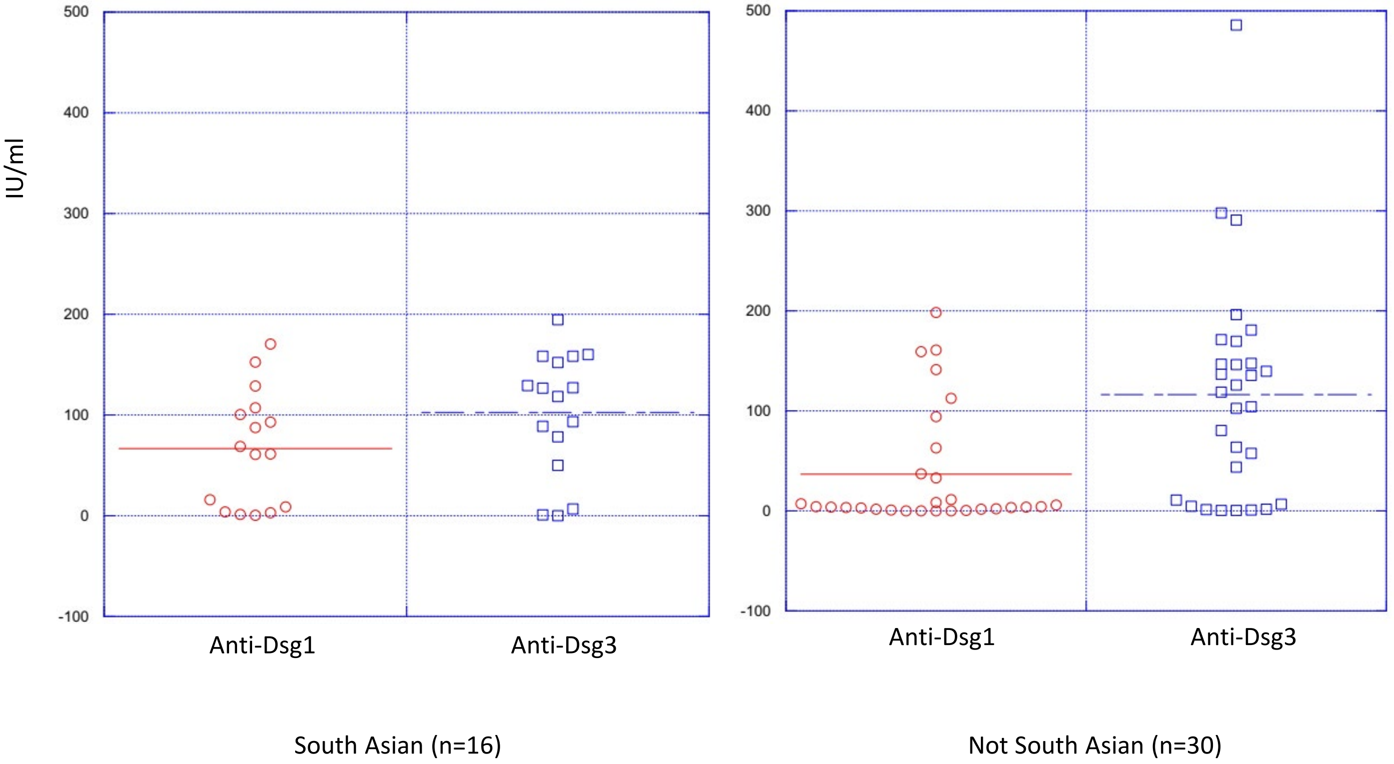
IU/ml
500
400
300
200
100
0
-100
Anti-Dsg1
Anti-Dsg3
South Asian (n=16)
500
400
300
200
100
0
-100
Anti-Dsg1
Anti-Dsg3
Not South Asian (n=30)
